# Local scale interventions dominate over catchment scale controls to accelerate the recovery of a degraded stream

**DOI:** 10.1371/journal.pone.0252983

**Published:** 2021-06-17

**Authors:** Alexander J. Sims, Ian D. Rutherfurd

**Affiliations:** 1 School of Geography, The University of Melbourne, Parkville, Australia; 2 Alluvium Consulting Australia, Cremorne, Australia; University of Bucharest, ROMANIA

## Abstract

A premise of stream restoration theory and practice is that it is often futile to attempt to restore a stream at the reach scale (10^1^–10^3^ metres) until catchment scale problems have been addressed. This study considers reach scale restoration actions undertaken in Bryan Creek, a sand bed river in south east Australia impacted by a sediment pulse, after catchment sediment sources have been addressed. Local scale interventions, which were in-stream sand extraction, fencing to exclude stock and riparian revegetation, were evaluated by quantifying cross-section and thalweg variability, mapping in-stream and riparian vegetation and by classifying the morphology that emerged following each intervention. Following intervention channel reaches moved to one of three distinct states: simple clay bed, eroding reaches dominated by *Juncus acutus*, and reaches with deep pools and *Phragmites australis*. Boundaries between the intervention reaches were sharp, suggesting local scale interventions dominate over catchment scale processes. The magnitude and spread of variability metrics were similar between all reaches and differences in variability bore no relation to intervention type, despite the stark difference in post-intervention morphology. These findings suggest that cross-section and thalweg variability metrics are an inadequate proxy for the effectiveness of local scale interventions in accelerating the recovery of sand bed reaches from a bedload pulse. The most important implications for river managers is that local scale interventions can lead to substantial and rapid improvements in condition, and the change in condition of these reaches is almost independent of other reaches. In this case, the key to the pattern of reach scale geomorphic recovery is excluding stock from waterways so that a specific macrophyte can establish, trap sediment and develop pools.

## Introduction

A premise of stream restoration theory and practice is that it is usually futile to attempt to restore a stream at the reach scale until basic problems in the catchment have been addressed [1–3]. This study considers the changes produced by reach scale restoration actions in the trunk stream after major catchment issues have been addressed. Restoration activities in those degraded trunk streams tend to involve multiple, diverse and disjointed interventions perhaps over hundreds of metres or a few kilometres of the stream and its riparian zone. This is because the stream is often owned by different landholders or managed by different groups. The messy reality of this sort of program of work is not amenable to controlled trials of effectiveness, which makes project evaluation very difficult [4, 5]. As a result, project evaluations tend to be similarly piecemeal or absent entirely. This study explores the outcomes of this type of piecemeal intervention in a stream affected by a pulse of bedload sediment.

Clearing of vegetation from catchment drainage lines and conversion to agricultural land use tend to increase erosion rates in upper catchments, which increases sediment supply through the stream network, which leads to formation of bedload pulses. Bedload pulses fill pools, bury in-stream wood and simplify the channel bed [6, 7]. The resulting channel is devoid of habitat and amenity value. The loss of channel capacity can also increase the frequency and duration of flooding [8–10]. Whilst such streams seldom return to their pre-disturbance state [11] they can recover some aspects of that state with time, and the recovery of such streams can be accelerated once the primary cause, the supply of sediment to the stream, has been addressed [10].

An important issue when assessing the success or otherwise of an intervention is what the treated reach is compared against. What constitutes a ‘recovered’ condition from a bedload pulse will vary with river type (sand vs. gravel bed streams), any limits on channel adjustment imposed by historic disturbances or interventions (e.g. recovery of a bedload pulse in a reach that was previously channelized), and with the hydrological and sediment regime now imposed on the river. In parallel to this issue of what constitutes a recovered state, is the challenge of how to objectively measure recovery. Metrics such as channel complexity, or a particular assemblage of landforms have proved useful [3, 12, 13], but the metrics are still contextual, relying on identifying the type and level of complexity a recovering channel is moving towards. Managers can set complexity targets by appropriately selecting reference reaches (which may be distinct from historical states of the river being treated), or by constructing conceptual models that are used to identify the morphologies possible for the target reach. Not all rivers are complex and not all ‘recovered’ states will be more complex than their degraded predecessors.

Recovery (in the limited, geomorphic sense we define it here) of streams impacted by a bedload pulse would be measured in terms of the return of pre-sand geomorphic structure [14, 15], an increase in the abundance and diversity of riparian and in-stream vegetation [16], an increase in the abundance and diversity of stream biota [17, 18], or an increase in the number and diversity of habitat units such as pools, stable bars and in-stream wood [19, 20].

This paper considers the effectiveness of several local scale interventions (10^1^–10^3^ metres in river length) implemented at the tail of a bedload pulse, when catchment scale erosion control measures have already been undertaken. The target stream is Bryan Creek, a tributary of the Glenelg River in south east Australia, and the interventions are sediment extraction, riparian revegetation, and stock exclusion. Clearing of vegetation and conversion to agricultural land use in the Bryan Creek catchment increased runoff and initiated widespread erosion and gullying. Increased runoff and sediment delivery caused Bryan Creek to incise, which transformed the creek from a chain of ponds to a continuous, eroding channel that then filled with sand as the increased catchment sediment supply led to the formation of a bedload pulse in the lower reaches of Bryan Creek [21]. The focus of this study is change in the physical structure of the channel (sediment, bars, floodplains features), and change in vegetation that occurred in Bryan Creek following interventions aimed at accelerating the recovery from the bedload pulse. Geomorphic complexity, the diversity of stable channel features and vegetation, is widely considered to be a crucial template for habitat, improved ecological condition and biodiversity [2, 13, 22–24]. Therefore, we use geomorphic complexity metrics to objectively quantify the post-intervention variability of Bryan Creek. We do not directly consider changes in aquatic biota.

The effect of an intervention would ideally be determined with a before-after-control-intervention (BACI) experimental design against reference and control reaches elsewhere [18, 25]. There are no appropriate reference or control reaches in this region, none were set-up at the time of the interventions, and many reaches of stream have multiple interventions. This is a common problem with post-hoc studies like this one. In this situation the best that can be done is to compare the response of reaches (direction and magnitude of change) to the response predicted by conceptual (or numerical) models, taking into account the interaction between reaches. We are fully aware of the limitations of this approach (especially the complex interactions between treated reaches), however, this is exactly the approach that managers take every day when planning projects such as these. They will assess the processes in the reach, predict the trajectory of change, the interaction between reaches, and so decide on how to intervene. This is, for example, the process when making a Riverstyles assessment [17, 26–28]. Therefore, we take the approach, in this study, of identifying a single river where local scale interventions are all subject to the same boundary conditions and environmental history. This approach is a feasible trade-off between an ideal study design with fully independent trials and controls, and the messy conditions likely to be present in most stream restoration projects.

## Bryan Creek study site

**Fig 1:**
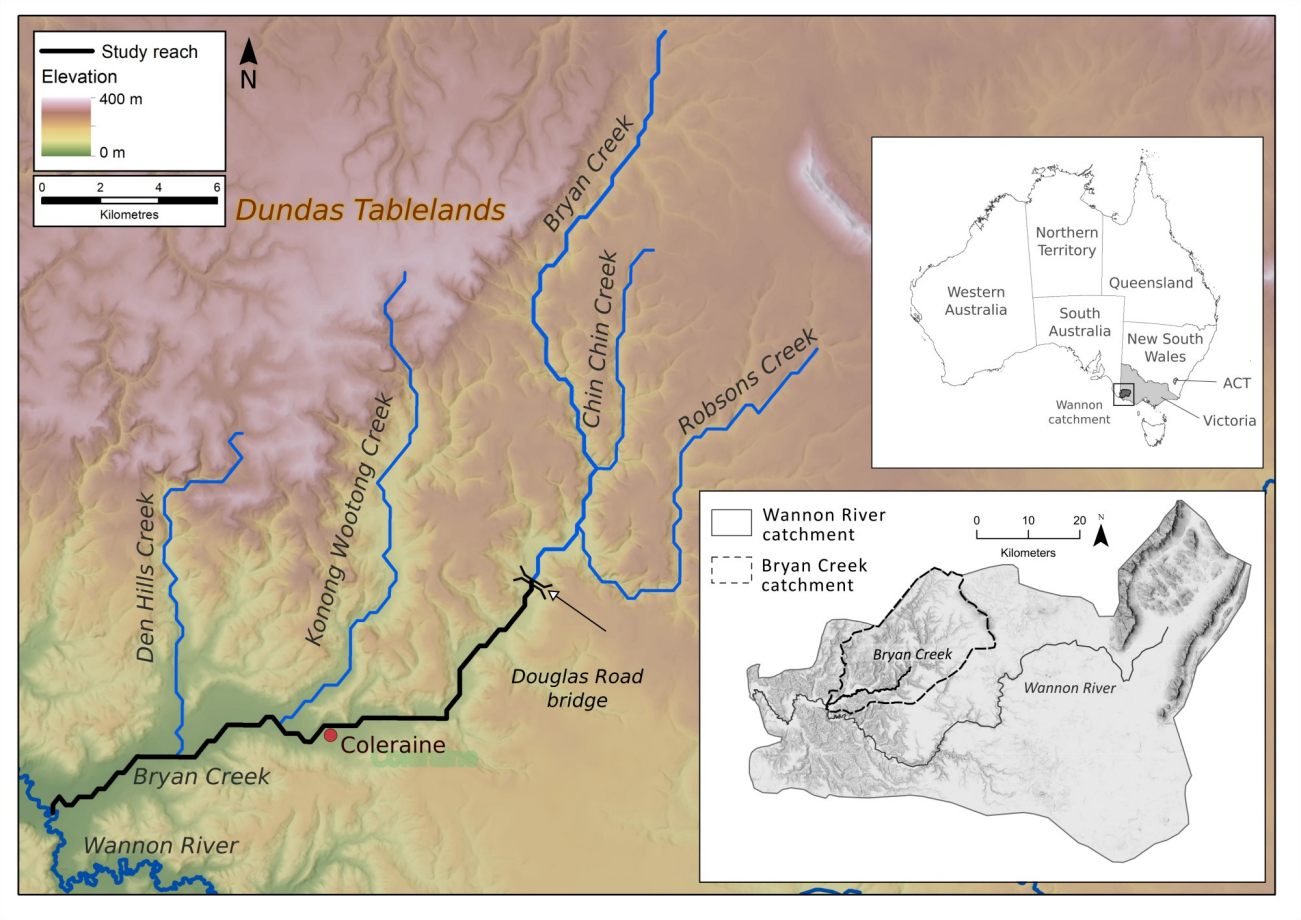
Bryan Creek catchment in western Victoria, Australia. The Wannon River junction and major tributaries are shown in blue and the study area is outlined in black.

Bryan Creek is a tributary of the Wannon River in western Victoria, Australia and has a catchment area of 560 km^2^. Here we focus on the lower 30 km, between the Douglas Bridge and Wannon River junction (Fig 1). The township of Coleraine is on the left bank in the middle of this reach.

Bryan Creek is an ideal case study because:

1. The supply of sediment from headwater gullies has declined, and the bedload pulse is now migrating downstream revealing a ~30 km stretch of river at the tail of the bedload pulse. Bryan Creek is a good example of the class of small, sand bed rivers in south east Australia [29] recovering from erosion induced bedload pulses where sediment supply from the catchment has declined.
2. It has experienced a full range of management interventions over several decades; sand extraction, stock exclusion and revegetation, but in different, distinct locations. This pattern of interventions allows us to compare the effectiveness of different interventions, alone and in combination. Further, the resulting pattern of change is remarkably distinct between these reaches, isolating the effect of local interventions.
3. There is a wealth of historical photography, channel surveys, and records of management actions, for the entire waterway over an unusually long period, 50 years.

The focus of this paper is on the recovery of a sand bed river from a bedload pulse. We summarise the catchment scale changes that drove pulse formation, the resulting changes in channel morphology of the study reach, and the type and location of management interventions later applied to Bryan Creek.

### Environmental history of Bryan Creek

**Fig 2:**
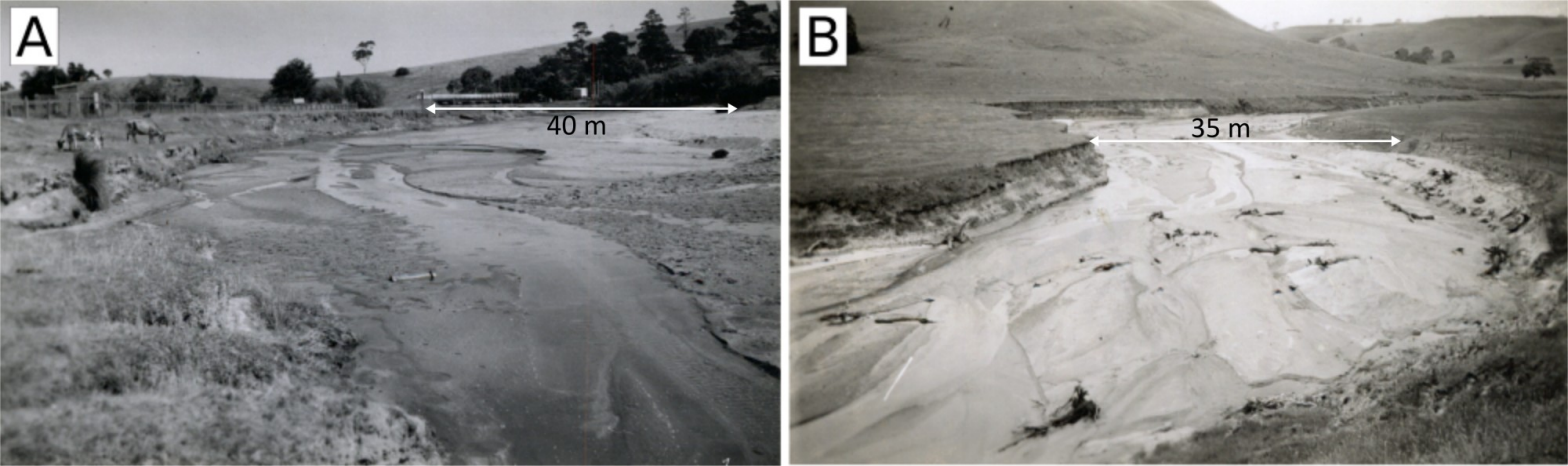
The sand pulse in Bryan Creek (A) looking downstream into the reach adjacent to the Coleraine Township in 1950, (B) looking upstream toward the reach immediately above Coleraine in 1944, substantial in-stream extraction was undertaken in this reach between 1954 and 1990. Photo source: Coleraine Historical Society.

In common with many south east Australian streams, at European settlement, Bryan Creek was a chain of ponds, which are intermittently spaced pools separated by densely vegetated channels [30–32]. Clearing of catchment vegetation in the early 19^th^ century across western Victoria [33] altered catchment hydrology and initiated widespread catchment erosion, gullying and stream incision [21, 34]. Bryan Creek first incised, and then filled with sediment (predominantly sand) as a bedload pulse formed. As the bedload pulse migrated downstream, aided by the flood of record in 1946 [35], the channel widened and the channel bed became a flat, featureless sheet of sand with no vegetation on the bed or banks (Fig 2A and 2B). No gauging stations are installed in Bryan Creek, but flows are typically higher in winter and may cease entirely in summer. The catchment is steep and quickly conveys runoff through the low gradient valley, where flows rework the coarse sands in the bed of Bryan Creek. The management interventions described in the following section were applied to this sand channel.

#### Erosion control measures in the Dundas Tablelands

**Fig 3:**
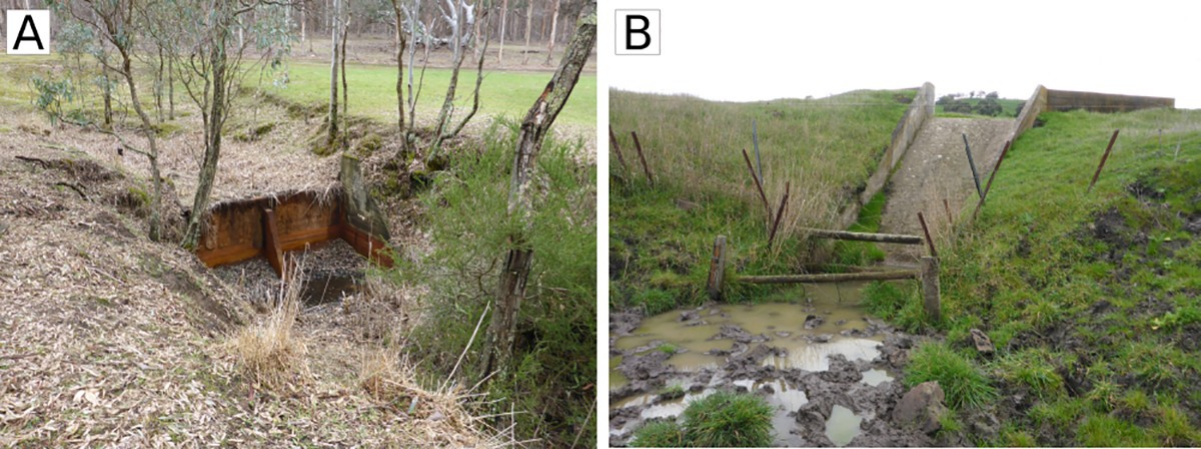
Erosion control structures, constructed in the late 1960s and early 1970s, in small headwater streams in the Bryan Creek catchment. (A) ‘Drop structures’–small grade control structures which span the channel and trap sediment, (B) concrete chutes which slow erosion by preventing nickpoints from migrating upstream. Photos courtesy of Glenelg Hopkins Catchment Management Authority.

**Fig 4:**
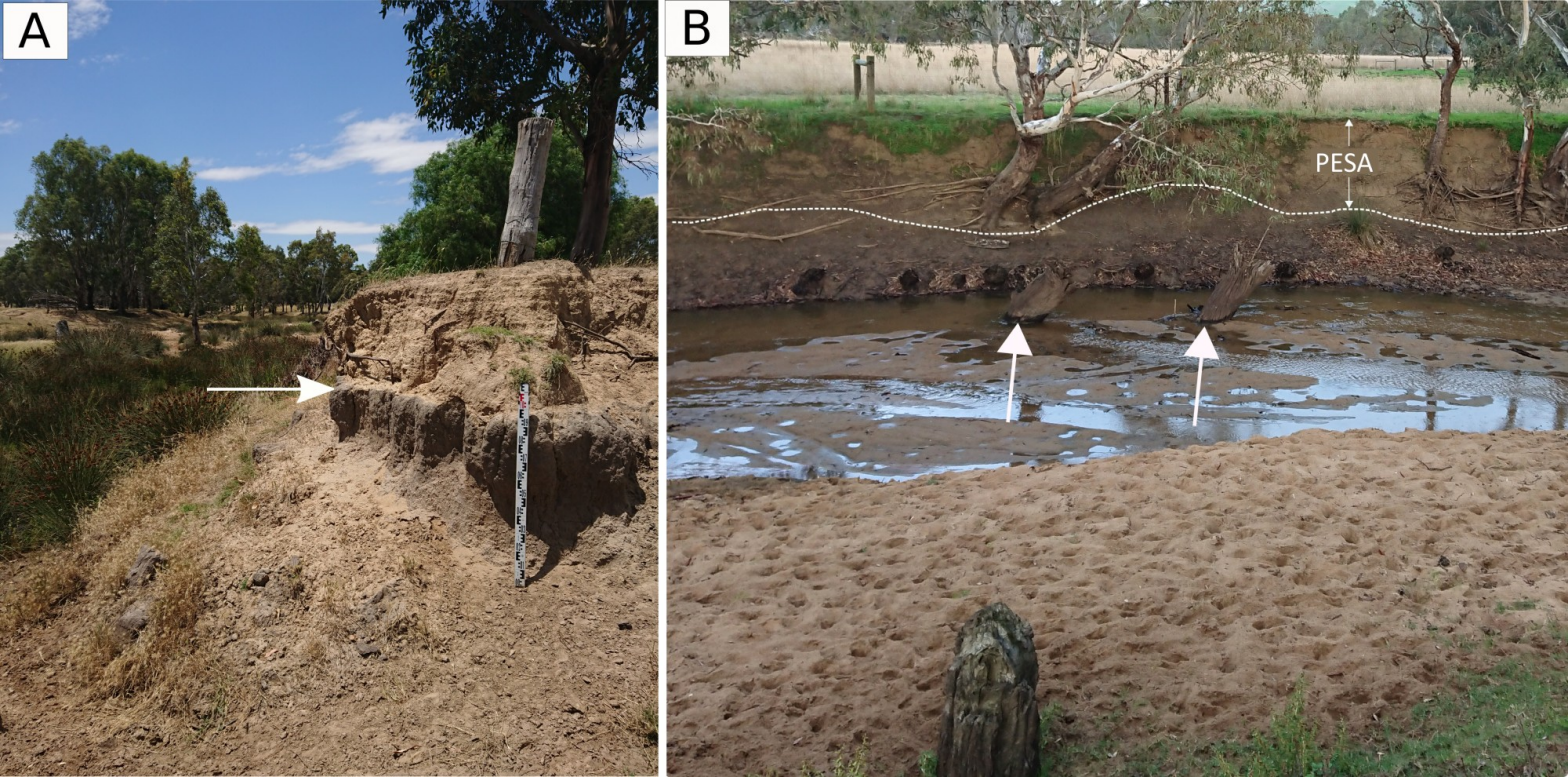
(A) Looking upstream into a reach not subject to any intervention, in 2017, the white arrow points to the sharp contact between the pre-pulse riverbank comprised of dark, organic rich sediments, and the upper levee of coarse sand deposited on the floodplain when the bedload pulse formed, (B) looking across towards the left bank in the lower part of Bryan Creek. Dashed white line marks the contact between the former stream bank and the overlying post-European settlement alluvium (PESA), white arrows mark exhumed tree trunks in the channel bed.

Erosion control works (fencing to exclude stock, wooden or concrete structures to halt gully-head migration, and grade control structures) were undertaken by the Soil Conservation Authority and Glenelg River Improvement Trust between 1950 and 1989 in the gullies that feed water and sediment to the study reach (Fig 3). These works have succeeded in stabilising gullies and have reduced sediment inputs to Bryan Creek [20, 21], allowing the tail of the sediment pulse to move downstream (Fig 4).

### The type and distribution of local scale interventions in Bryan Creek

**Fig 5:**
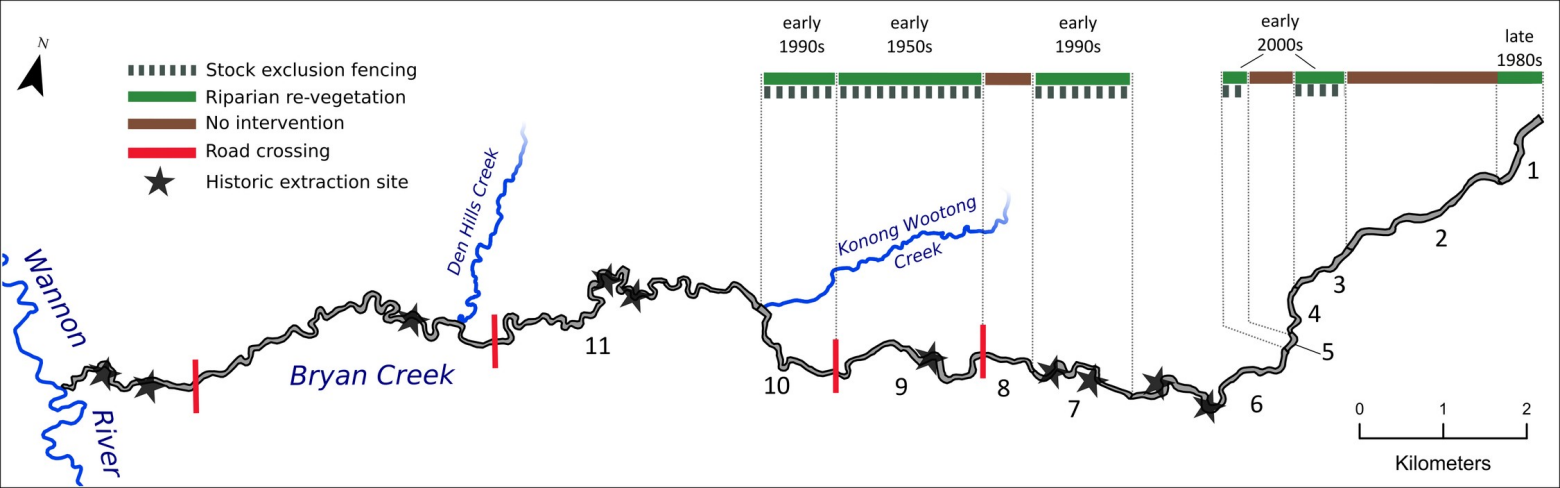
Study reach of Bryan Creek in western Victoria with treatment reaches numbered. Coloured bars indicate the type of intervention undertaken in the corresponding reach and the approximate date of intervention. The location of extraction sites (1954 to 1994) and road crossings are also shown. Flow is from right to left.

Since the 1980s stock exclusion fencing, revegetation and in-stream sand extraction have been used in five combinations, across eleven distinct reaches of Bryan Creek (Table 1 and Fig 5).

**Table 1:** The combination of intervention used, the corresponding number of reaches treated, and the cumulative length of river treated by each combination of intervention.

Combination of interventions	Number of reaches	Combined stream length (km)
Fence and revegetate	3	2.5
Revegetate only	1	0.9
Sand extraction only	2	16.5
Fence + revegetate + extraction	2	4.2
No treatments	3	3.3
**Total**	**11**	**27.5**

**Fig 6:**
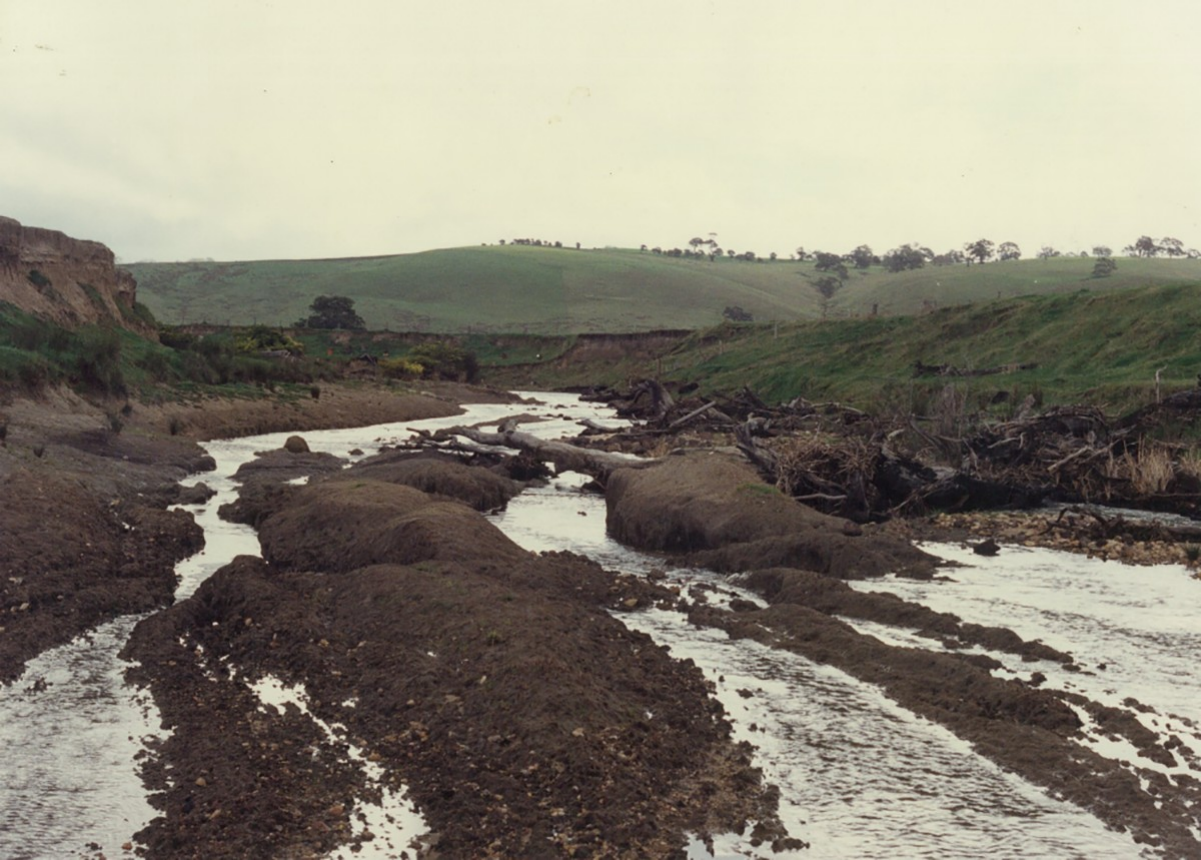
Looking downstream into the clay bed reach at the upstream end of the study reach (reaches 1 and 2 in Fig 5) immediately prior to intervention in the late 1980s, twenty years after erosion control measures and improved pasture practices in the catchment reduced the supply of sediment to Bryan Creek. Note incision into the clay bed. Photo source: Glenelg Hopkins Catchment Management Authority.

**Fig 7:**
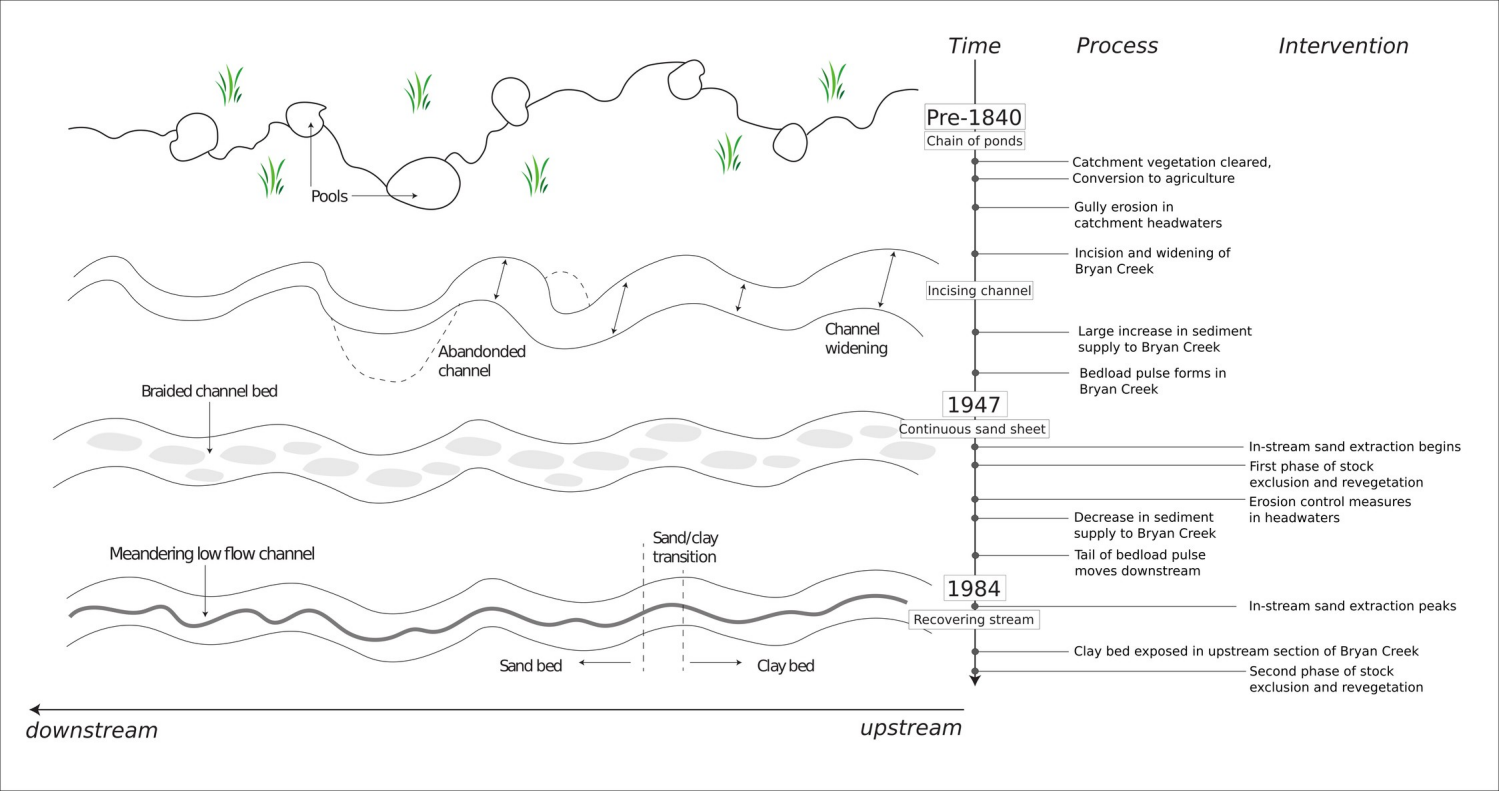
Changes in the catchment of Bryan Creek, the response of Bryan Creek to catchment changes and the timing of intervention between 1950 and 1984.

The following descriptions refer to the reaches shown in Fig 5. In-stream sand extraction was undertaken continually, but in different parts of the river, between 1954 and 1994. Sand was extracted from discrete pits approximately 40 m in length, 30 m wide and 3–4 m in depth, or in the case of reach 7, from a much larger zone approximately 100 m long and 40 m wide. Stock exclusion, which consisted of fencing both sides of the river, and revegetation, which consisted of planting trees on both banks of the river, but not on the streambed, was undertaken in reach 9 first, in the 1950s. When the second phase of interventions began in the 1980s, the tail of the bedload pulse had moved further downstream, exposing a clay bed in the upper 3 km of the river. Thus, when stock exclusion and revegetation treatments were undertaken in reach 1 (Fig 6), the reach had an incising, clay bed. Reaches 7 and 10, which were sand bed reaches at the time of intervention, underwent stock exclusion and revegetation in the early 1990s. Lastly, reaches 3 and 5 (also sand bed reaches at the time of intervention) were fenced to exclude stock and had their banks revegetated in the early 2000s. Fencing in reaches 3 and 5 was not maintained, but stock exclusion fencing in other reaches was. The impact of catchment scale changes in vegetation cover and sediment supply on the morphology of Bryan Creek, and how the timing of these changes relates to the local scale interventions just described, are summarised in Fig 7.

## Methods

We evaluate the impact of local scale interventions implemented once the catchment scale drivers of degradation have been addressed by separating Bryan Creek into 11 reaches, according to the extent of different local scale interventions. Here we define a *reach* as a distinct length of stream that experienced the same set of interventions.

### Study design

For this study we exploit the well documented history of Bryan Creek, a valley floor incised stream in western Victoria, Australia which is recovering from a large bedload pulse. We use the history of change to assess the consequences and interactions of three common, local scale interventions: fencing to exclude stock from the channel bed and banks, revegetation of the banks, and commercial sand extraction to starve downstream reaches of sediment.

Although our focus is geomorphic rather than ecological changes, we recognise that interventions to promote the former are often part of wider strategies to improve stream health. Thus, we also evaluate whether interventions increase geomorphic complexity, a widely employed surrogate for geomorphic condition and habitat availability [12, 13, 22, 36]. Finally, we characterise the pre-intervention morphology of Bryan Creek and assess how this morphology changed following each local scale intervention.

### Channel surveys

We generated a baseline longitudinal profile by compiling a total of 98 cross-sections surveyed in 1984 at variable spacing (50 m– 1 km) along the study reach, and then extracting the thalweg of each cross-section. The baseline survey data does not cover a portion of the study reach near Coleraine and many of the cross-sections were captured with very few elevation measurements across the stream. A post-intervention longitudinal profile was constructed by surveying a total of 91 cross-sections using a Trimble R6 differential GPS during a 2017 field survey. The average point-spacing of elevation points in each cross-section was 2 m, and the average spacing between cross-sections was 50 m. GPS cross-sections were surveyed in dense vegetation or standing water, areas unlikely to generate accurate return in an upcoming (2019) aerial LiDAR survey. LiDAR was flown in 2019 and returns had a vertical and horizontal accuracy of ± 0.1m. LiDAR returns were interpolated to generate a 1m x 1m DEM. Cross-sections were then generated in areas of the stream bed with exposed sand at a spacing of 50 m using a GIS, before elevation points were extracted along each cross-section (average point spacing 2m). The 2019 LiDAR cross-sections, and the 2017 GPS cross-sections were then merged to produce a total of 578 cross-sections, spaced at 50 m intervals along the river. Thalwegs were extracted from these post-intervention cross-sections.

The 1984 and 2017/2019 cross-sections were referenced to common chainage (originating at the junction of Bryan Creek and the Wannon River) to enable clear comparison between pre and post-intervention longitudinal profiles. Both profiles were de-trended by modelling thalweg elevations with a second order polynomial equation fit to a chainage vs. elevation plot of the baseline profile. A smooth polynomial, rather than a linear equation, was used to de-trend the profiles so that all residual thalweg elevations would be centred on a value of 0. Modelled thalweg elevations were subtracted from the surveyed thalweg elevation values to generate a plot of residual thalweg elevation vs chainage for both profiles.

Bankfull channel width (maximum cross-section width) and channel depth (the difference between the left bank elevation and the thalweg elevation in each cross-section) were calculated for all point in all of the 2017/2019 cross-sections. The outcome of this step was a detailed dataset of cross-section depths and thalweg elevations for the post-intervention channel.

### Geomorphic complexity metrics

To supplement our subjective classification of morphology, and to objectively compare the geomorphic condition of reaches subjected to different management interventions, we employed quantitative metrics of geomorphic complexity [12, 13, 37].

Bartley and Rutherfurd [12, 20] assessed the ability for different variability metrics to distinguish between degraded and recovering reaches in the Wannon River and found that the standard deviation of cross-section depths, vector dispersion and squared height differences captured a wide range of channel shapes and variability. We adopt these same metrics to quantify complexity in Bryan Creek (Table 2).

**Table 2:** Variability metrics used in this study.

Variability metric	Formula	Description of calculation
Standard deviation of depths	$\sigma =\frac{\sqrt{\frac{\sum {\left(x_{i}-\mu \right)}^{2}}{N}}}{WP}$ Where *σ* is the standard deviation of depth, *x*_*i*_ is the depth at a point along the transect, *μ* is the mean depth of all point along the transect and *WP* is the wetted perimeter of the transect.	Normalized by the wetted perimeter of cross-sections or thalwegs to account for varying cross-section widths and reach lengths. Standard deviation of depth values for thalwegs were multiplied by 10 to bring them into the same order of magnitude as the other metrics.
Vector dispersion	$VD=\frac{\left(n-[\sum_{i=1}^{n}\left(\frac{A}{C}\right)]\right)}{n-1}$ Where VD is the vector dispersion metric, *n* is the number of points along the transect, *A* is the is the distance between points and *C* is the distance along the bed.	A measure of angular variability (between successive points in a cross-section or longitudinal profile) which takes account of cross-section width and profile length within the calculation. Vector dispersion values of cross-sections were multiplied by 100 to bring value into the same order as magnitude as the other metrics.
Sum of squared height differences	$SSh=\frac{\sum dh^{2}}{WP}$ Where *SSh* is sum of squared height differences, *dh* is the change in elevation between successive points along the transect and *WP* is the wetted perimeter of the transect.	Normalized by the wetted perimeter of cross-sections or thalwegs to account for varying cross-section widths and reach lengths.

Metrics were drawn from [12], which evaluated complexity metrics in sand bed streams impacted by sediment pulses.

Baseline cross-sections were surveyed with few points in each cross-section, and cross-sections were spaced too far apart for meaningful estimates of cross-section or thalweg variability to be calculated. Therefore, we apply our complexity metrics to each of the post-intervention cross-sections and thalweg profiles of each reach.

### Changes in channel morphology

Changes in channel morphology were assessed by compiling available aerial photography for the study reach, which was captured in 1947 (B&W and at low resolution), 1963 (B&W), 1975 (B&W), and 1991 (colour). The final post-intervention morphology was captured using a combination of field inspections completed in 2017, and analysis of 0.5 m resolution aerial imagery captured in 2019. A small UAV was used during the 2017 field inspection to capture 0.05 m resolution aerial images in selected areas and to aid interpretation. We then used our field inspections and aerial imagery to develop a simple post-intervention morphological classification. The classification considers: the condition of the bed (the number and size of in-channel features such as bars, benches and pools and the presence and planform of the low flow channel), condition of the banks (whether banks were actively eroding or stable), and the abundance and types of vegetation.

We then compared the mapped extent of each intervention with our morphological classification to establish the spatial and temporal association between intervention type and morphology classification. The change in morphology at the boundary between reaches (no change, gradual or abrupt) was noted.

### Changes in vegetation

We used colour aerial imagery captured in 1991 and 2019 to map in-stream vegetation within each of the 11 reaches so that vegetation coverage could be compared to intervention type. To account for the different length of each reach, total vegetation coverage (m^2^) was converted to percentage coverage per metre of stream length using the equation:

$${Veg}_{cov}=\left(\frac{A_{veg}}{R_{L}}\right)\times 100$$

Where *Veg_cov_* is coverage of vegetation per metre of stream length (m^2^/m), *A*_*veg*_ is the total area of vegetation on the bed (m^2^) and *R*_*L*_ is the length of the reach (m). Vegetation mapped in the coloured aerial imagery was partitioned among the species already identified during the 2017 field surveys.

## Results

We outline changes in Bryan Creek generally, changes in vegetation, our complexity metrics, the results of our morphological classification and how channel morphology relates to the different combinations of local intervention.

### General channel changes

Overall, the flat sheet of sand present in 1947 steadily transitioned to a more complex, well-defined, meandering low-flow channel; but this change occurred at different times in different reaches. It is interesting to note that, although stock were excluded, and the banks revegetated from reach 9 thirty years earlier than other reaches, accelerated morphological changes began at the same time for all reaches in the early 1990s. This change coincides with the end of in-stream extraction and the expansion of emergent macrophytes into the channel.

### Changes in vegetation

**Fig 8:**
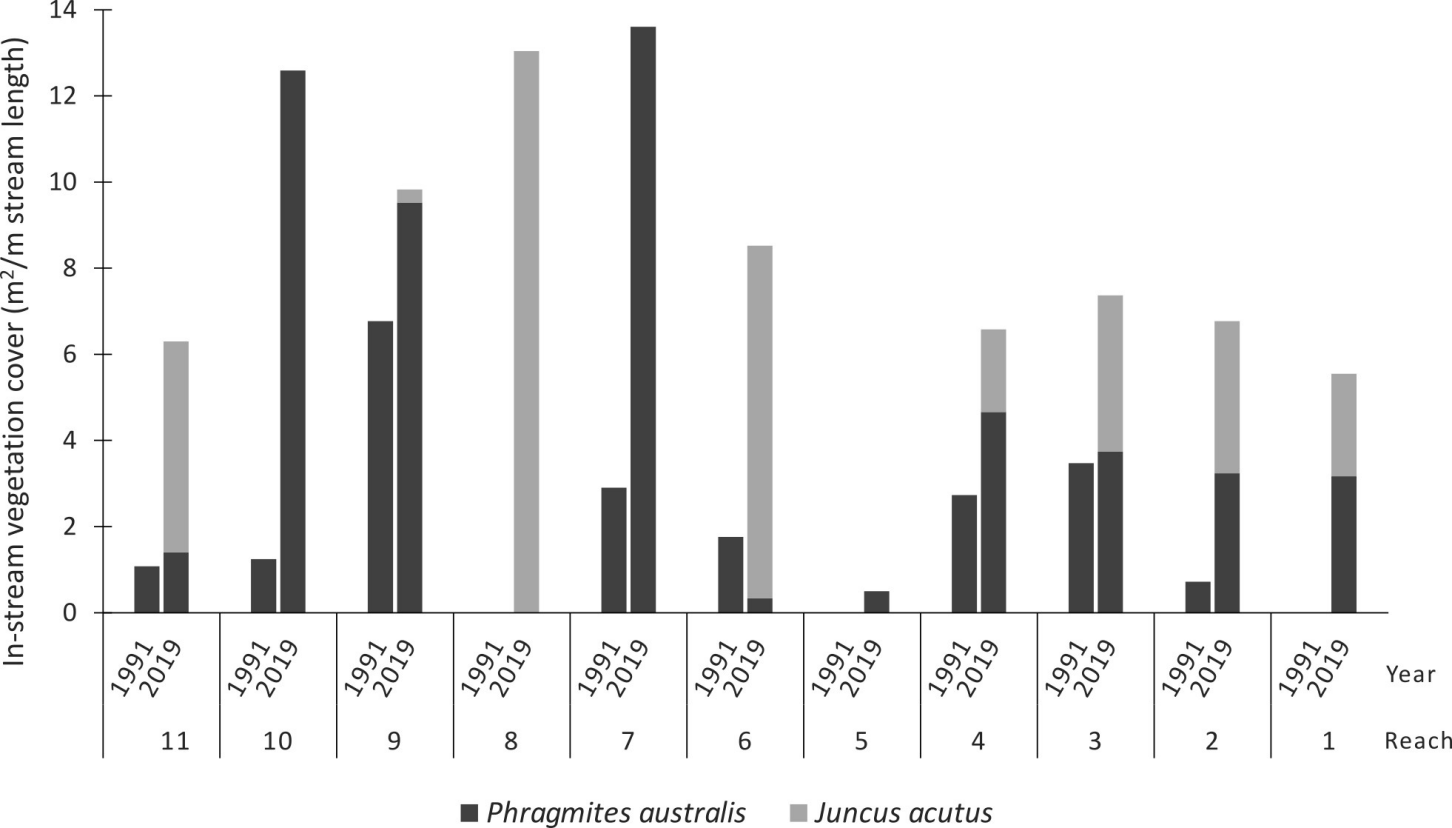
Changes in the coverage and compassion of vegetation in the 11 reaches of Bryan Creek between 1991 and 2017.

Vegetation coverage increased in all reaches between 1991 and 2019, but the species responsible for the increased coverage varied between reaches (Fig 8). Reaches 1–3 transitioned to an approximately even coverage of *Phragmites* and *Juncus*. The coverage in reaches 6, 8 and 11 was due almost entirely to *Juncus*, while increased coverage in reaches 7, 9 and 10 was due entirely to *Phragmites* (Fig 8). Reach 5 had the smallest increase in coverage, all of which was *Juncus*. Overall, reaches treated with stock exclusion fencing that was maintained are now dominated by *Phragmites* only, while those that have not had stock excluded or stock exclusion fencing was not maintained, have significant beds of *Juncus* throughout.

### Complexity metrics

#### Changes in the longitudinal profile of Bryan Creek

**Fig 9:**
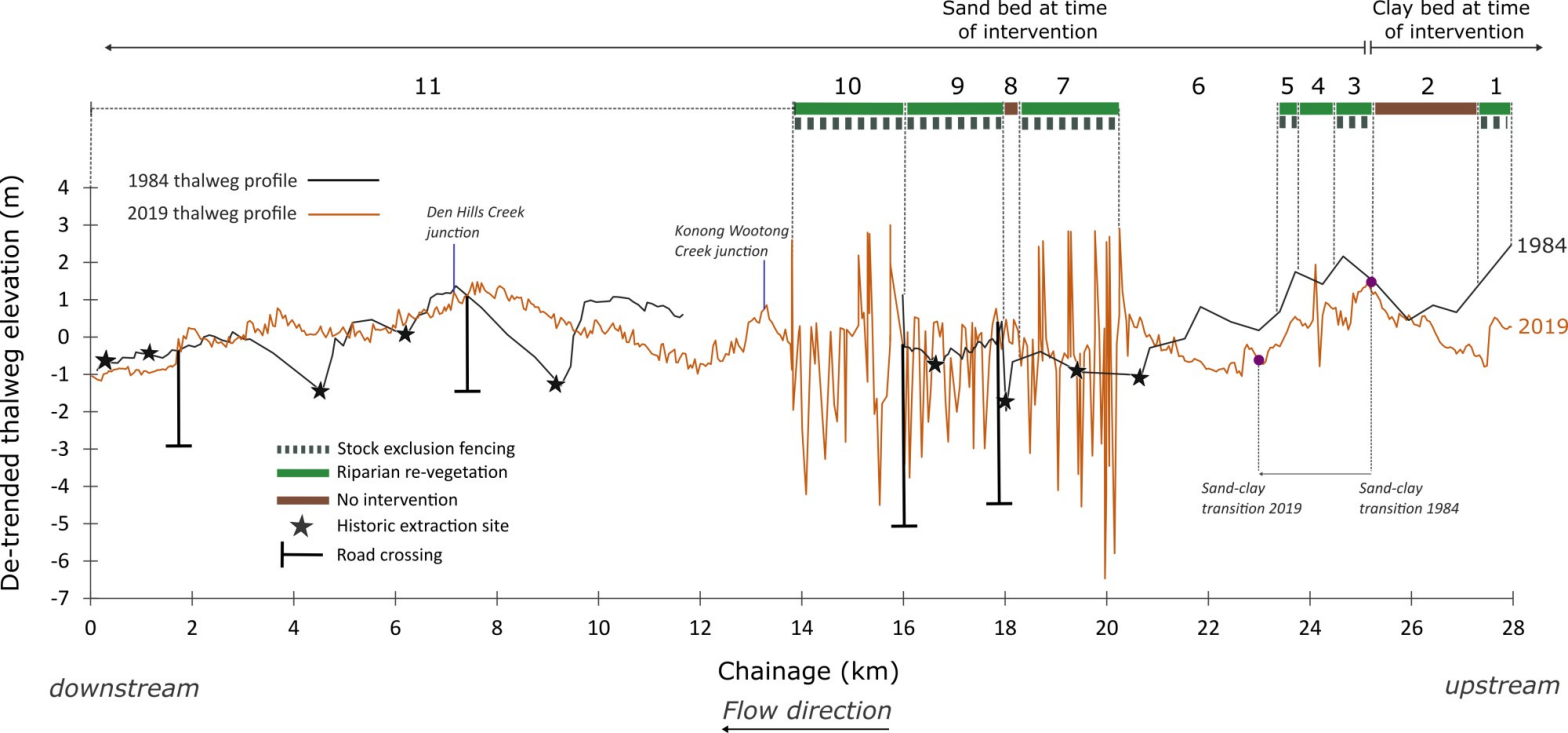
De-trended, baseline and post-intervention longitudinal thalweg profiles for Bryan Creek. Black line is the 1984 profile and orange line is the 2019 profile. The eleven intervention reaches, the combination of interventions undertaken in each reach and channel substrate at the time of intervention are annotated. The location of historical extraction sites is marked with black stars (each star is a single extraction pit). The position of the sand-clay transition in 1984 and 2017 is also marked.

Between 1984 and 2019 the tail of the bedload pulse (identified as the transition between sand and the clay on the stream bed) has continued to move downstream. This has caused the bed to degrade and has exposed the clay in reaches 3,4,5 and part of reach 6 (Fig 9).

The central portion of the river has aggraded overall (reaches 7–9). Aggradation in reach 11 is centred on the site of former extraction pits, while degradation has occurred at the upstream and downstream segments of the reach.

The most dramatic change in the river has been the development of deep pools in reaches 7,9 and 10 in the central section of the river. In these reaches, stock have been excluded by fences and the banks have been revegetated (Fig 5). In these reaches the thalweg has developed by degrading (eroding pools) and aggrading between the pools.

**Fig 10:**
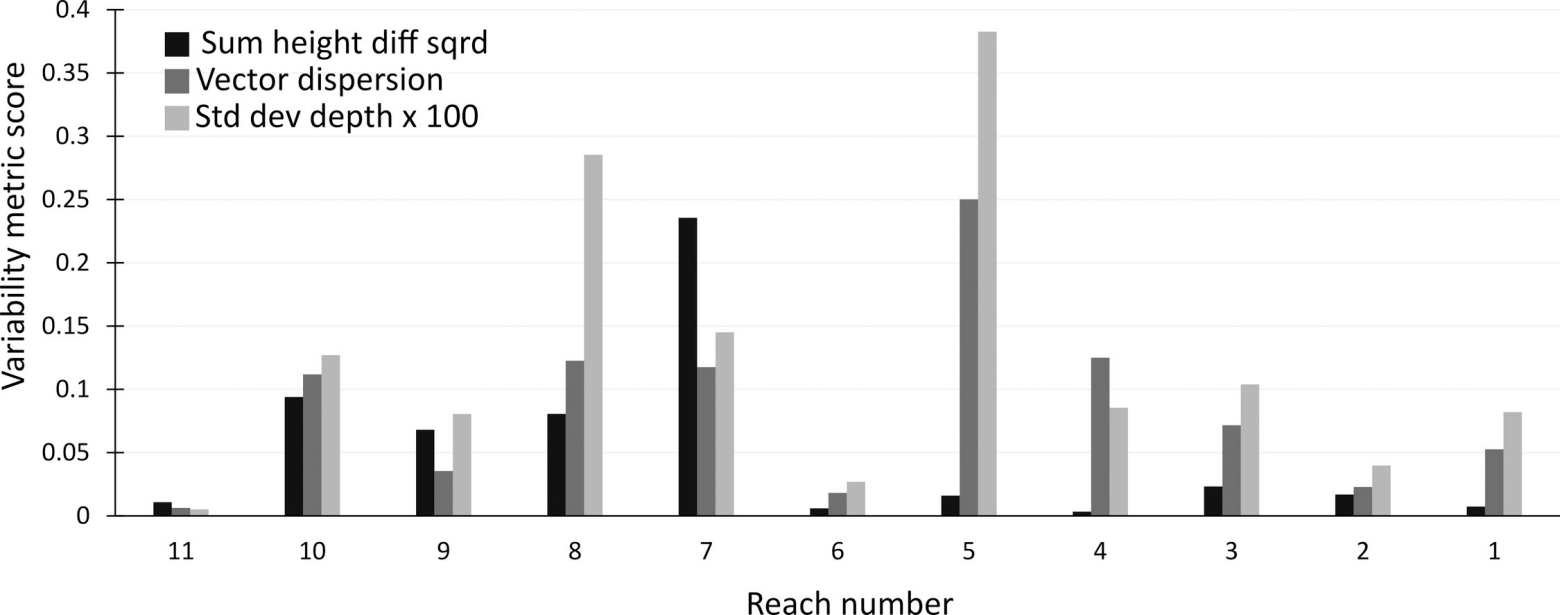
Post-intervention thalweg complexity values measured using the std dev depths, vector dispersion and sum height diff squared metrics for each of the 11 reaches of Bryan Creek.

Thalweg complexity values for all metrics are low in reaches 1,2,3, 6 and 11 (Fig 10). Values of sum height diff squared are consistently higher in reaches 7–10 (which mirrors the development of pools evident in the thalweg profile), and to a lesser extent so are values of vector dispersion and std dev depth. Reach 5 has notably higher values for std dev depths and sum height diff squared than surrounding reaches, but there is considerable overlap in calculated values of variability between reaches (Fig 10).

#### Cross-section variability

**Fig 11:**
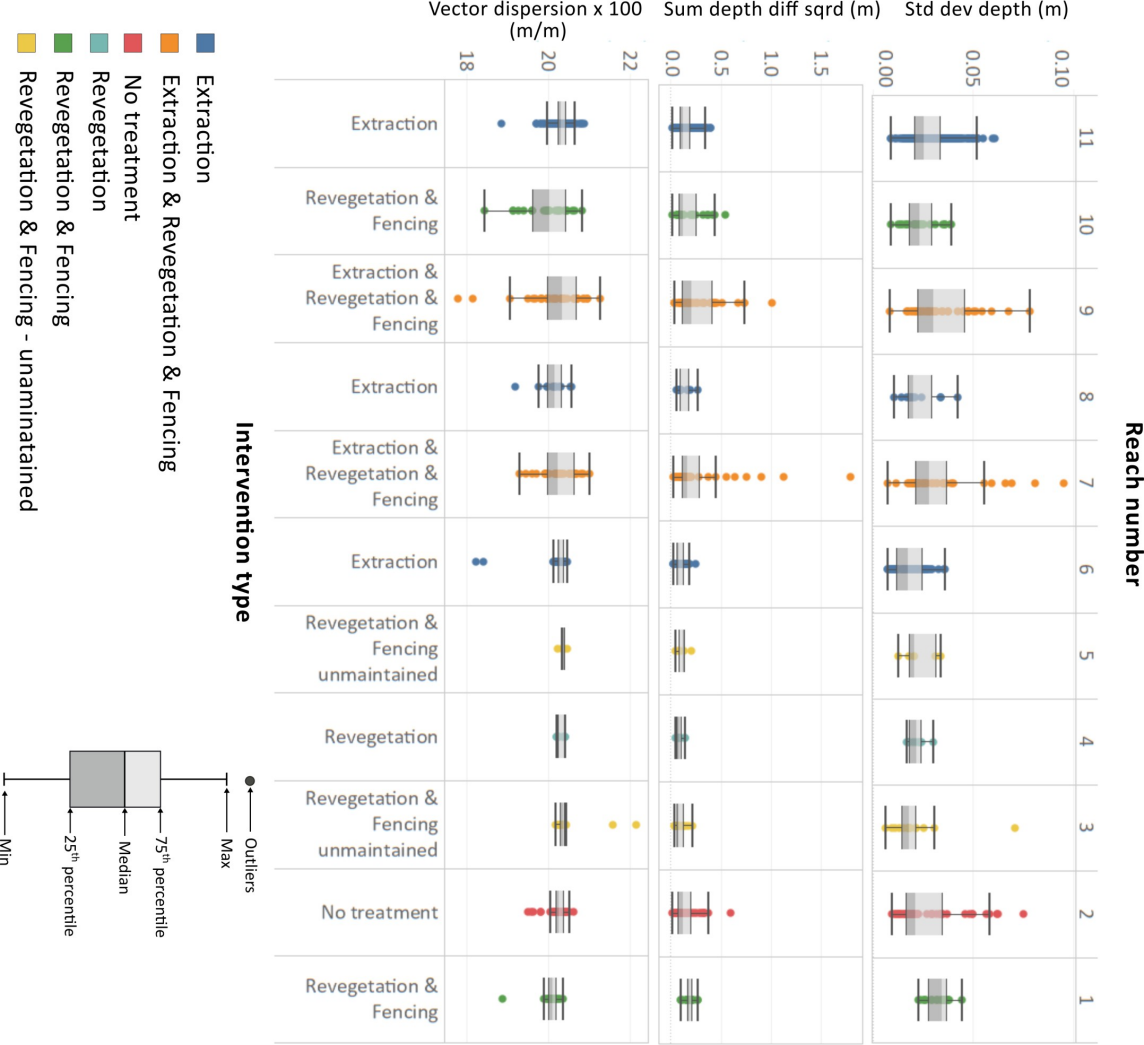
Cross-section complexity values for the std deviation of depths, sum of depth differences squared and vector dispersion metrics for the 11 reaches of Bryan Creek. Colours and labels at the base of the plot denote the intervention applied to each reach. Note that max/min values in box plot are defined as the 25^th^/75^th^ percentile values ± 1.5 x the inter-quartile range.

Cross-section variability does not show a clear pattern at the scale of the whole river (Fig 11). There is considerable overlap in the spread (as measured by the inter-quartile range) of cross-section variability values between reaches, regardless of the metric used to measure variability (Fig 11). However, reaches 7–11 have a wider distribution of variability than reaches 1–6, with reaches 7 and 9 having an especially wide range of variability values for all three metrics (Fig 11).

### Classification of reach scale morphology

In the last section we described the changes in cross-sectional and longitudinal changes in the 11 reaches. The abrupt changes in longitudinal thalweg variability are associated with changes in reach morphology. Post-intervention channel morphology varied by the type of intervention applied and channel substrate at the time of intervention (sand vs. clay) (Table 3).

**Table 3:** Width-depth ratio, thalweg variability and cross-section variability grouped by intervention type, for each of the eleven reaches in Bryan Creek.

Reach	Treatment type (substrate at time of intervention)	Median w/d ratio in 2019(std dev)	2017 channel morphology
1	Revegetation only (clay bed)	14 (3)	Simple
2	No treatment	17 (12)	Simple
3	Unmaintained stock exclusion and revegetation (clay bed)	27 (16)	Simple
4	No treatment	23 (5)	Simple
5	Unmaintained stock exclusion and revegetation (sand bed)	19 (5)	Simple
6	Extraction only (sand bed)	24 (14)	Dissected
7	Extraction then stock exclusion and revegetation (sand bed)	20 (8)	Sequence of pools
8	No treatment	27 (7)	Dissected
9	Extraction then stock exclusion and revegetation (sand bed)	21 (10)	Sequence of pools
10	Stock exclusion and revegetation (sand bed)	26 (8)	Sequence of pools
11	Extraction only (sand bed)	18 (7)	Dissected

The spatial extent of each intervention type is shown in Fig 9.

**Fig 12:**
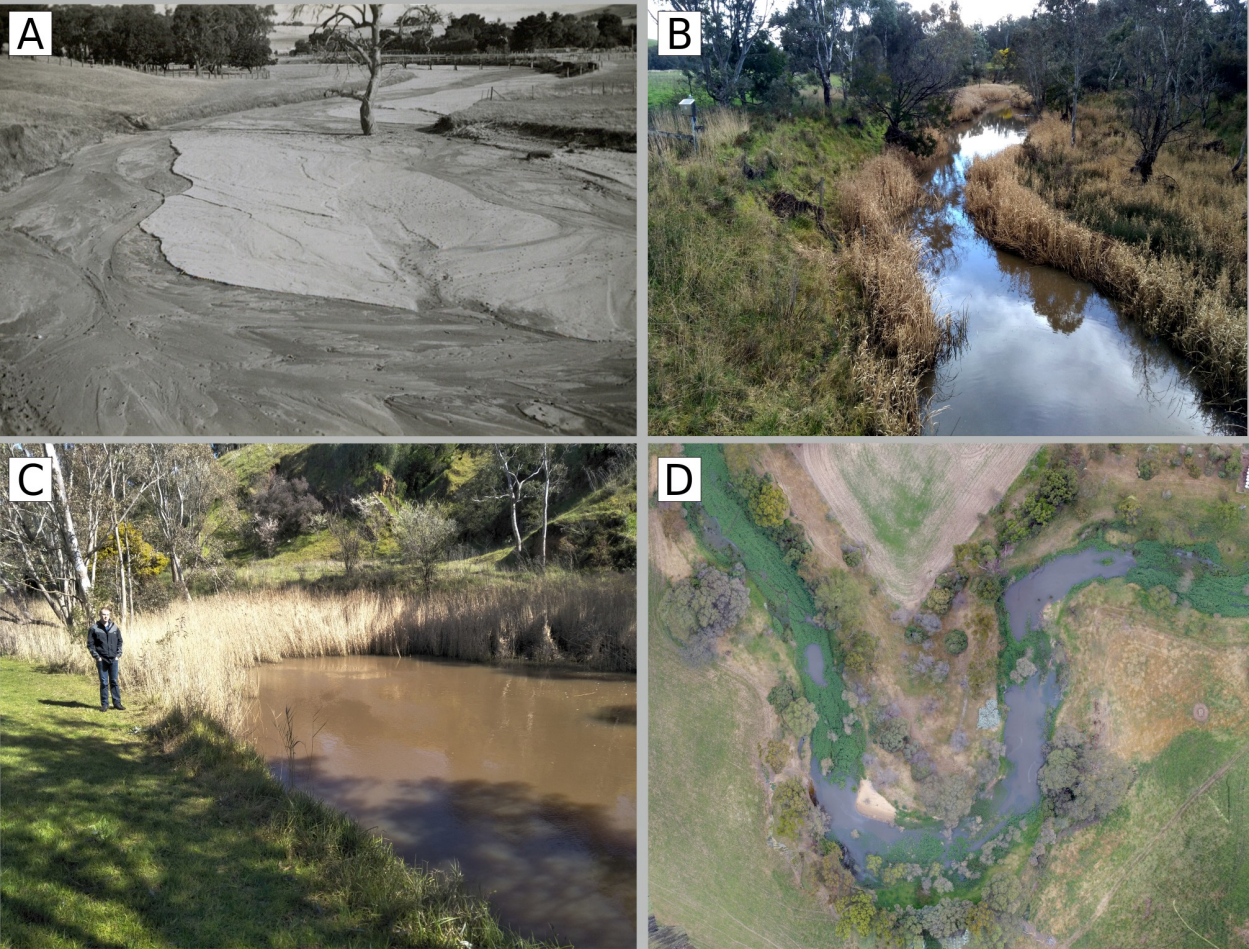
(A) View looking upstream into reach 10 beside the Coleraine township in 1940, showing wide, flat bed of sand devoid of vegetation, (B) looking downstream from the upstream end of reach 8 in 2017 showing emergent macrophytes on the margin of a continuous channel, (C) pools in roughly the same location as (A) but looking downstream in reach 10 in 2017, (D) a 2017 aerial image of reach 11 showing the sequence of pools. Photo A is from the Coleraine Historical Society.

**Fig 13:**
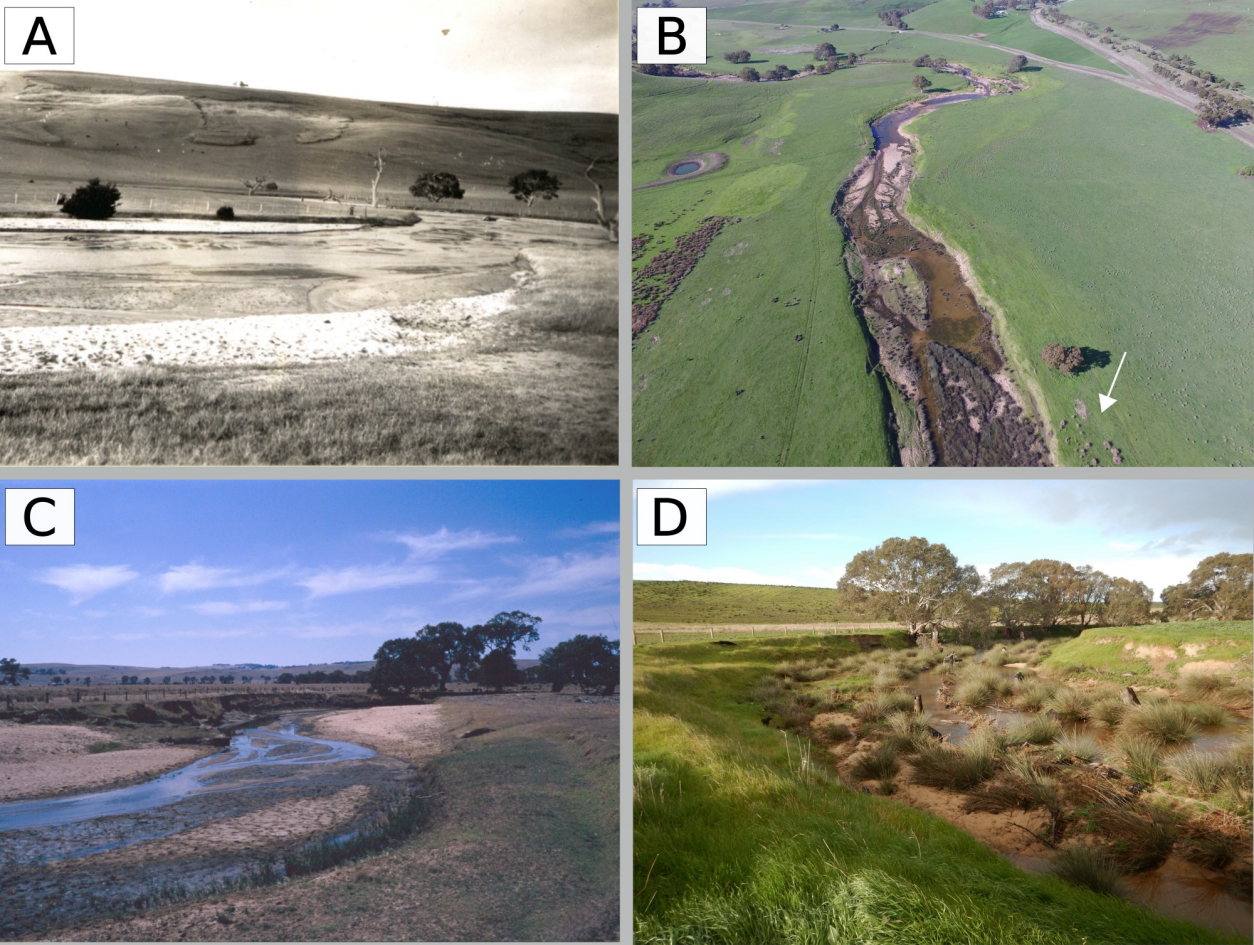
(A) Looking north across reach 7 above Coleraine in 1944, significant in-stream extraction began here in 1954, (B) aerial view of the same section in 2017 showing dissected morphology, white arrow indicates the position photo (A) was taken from, (C) representative section of reach 11 in 1994, immediately after extraction ceased, note the absence of *Juncus* on the channel bed, and (D) representative section of reach 11 in 2017, showing *Juncus* colonising the bed, the dissected morphology, and bar-and-bench erosion. Photo source: A, Coleraine Historical Society, B, Glenelg Hopkins Catchment Management Authority.

**Fig 14:**
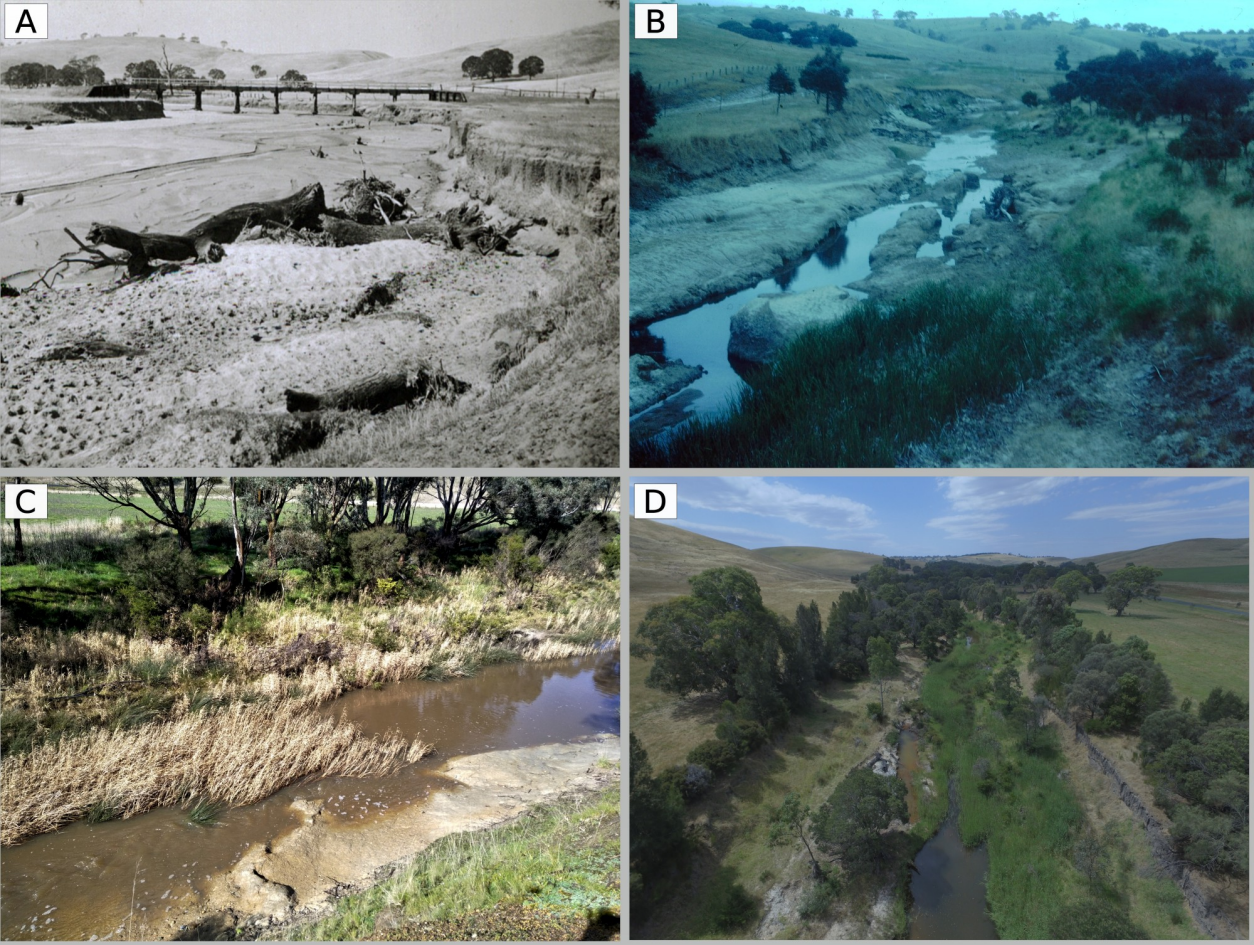
(A) Reach below original Douglas-Gaze-McMahon Road bridge, looking upstream, in 1940, (B) reach 1 looking upstream towards the current Douglas Road bridge, which is 300 m upstream from the original bridge, in the late 1980s, incising clay bed and bank-collapse clearly visible, (C) reach 1 in 2017, looking across from the right bank showing revegetated banks, small stands of *Phragmites* and exposed clay bed, (D) oblique aerial view of reach 1 looking upstream, showing formation of a series of shallow runs, emergent macrophytes and bank vegetation. Photo source: A, Coleraine Historical Society, B, Glenelg Hopkins Catchment Management Authority.

The characteristics of all 11 reaches can be classified into one of three distinct channel types:

- **Sequence of pool**: channels that alternate between dense stands of emergent macrophytes (*Phragmites australis*) and pools (~2–4 m deep), and well vegetated banks of mature red gums (*Eucalyptus camaldulensis*) (Fig 12B–12D).
- **Dissected**: a network of narrow, low-flow channels cut through beds of *Juncus acutus* and occasional clusters of emergent macrophytes (*Phragmites australis*), some bench development and pervasive erosional features such as bar cut-offs and near vertical, exposed banks (Fig 13B and 13D).
- **Simple**: channels that alternate between dense stands of emergent macrophytes (*Phragmites australis*), long, shallow pools (~0.5 m deep) and wide, flat sections with exposed clay, sporadic in-channel vegetation and high, steep banks (typical examples shown in Fig 14C and 14D).

We also note that in the section of Bryan Creek upstream of the Douglas Road bridge (outside our study area), clay has been exposed in the channel bed since at least 1984, with the 2017 morphology in this upstream section being similar to our *simple* classification.

We now consider the association between the combination of local scale interventions applied and the morphology that has developed.

### Association between intervention type and morphological classification

Reaches with a clay bed (reaches 1–5), either pre-intervention or where the clay bed has since been exposed, have transitioned to a simple morphology, regardless of which interventions (if any), were applied to them (Fig 14). In reach 1 this transition was preceded by a phase of deep incision, evident by the 1947 and the early 1980s photograph looking upstream (Fig 14), while in reaches 2–5 incision was similarly intense but has occurred since 1984 (Fig 9).

Reaches with a sand bed in 2017 (6–11) have transitioned to either a sequence of pools morphology (Fig 12) or a dissected morphology (Fig 13). The sequence of pools morphology has formed wherever stock have been excluded from a reach. All reaches treated with stock exclusion also had their banks revegetated, and the association between stock exclusion and sequence of pools morphology holds where the reach was previously subject to in-stream extraction (reaches 7 and 9).

**Fig 15:**
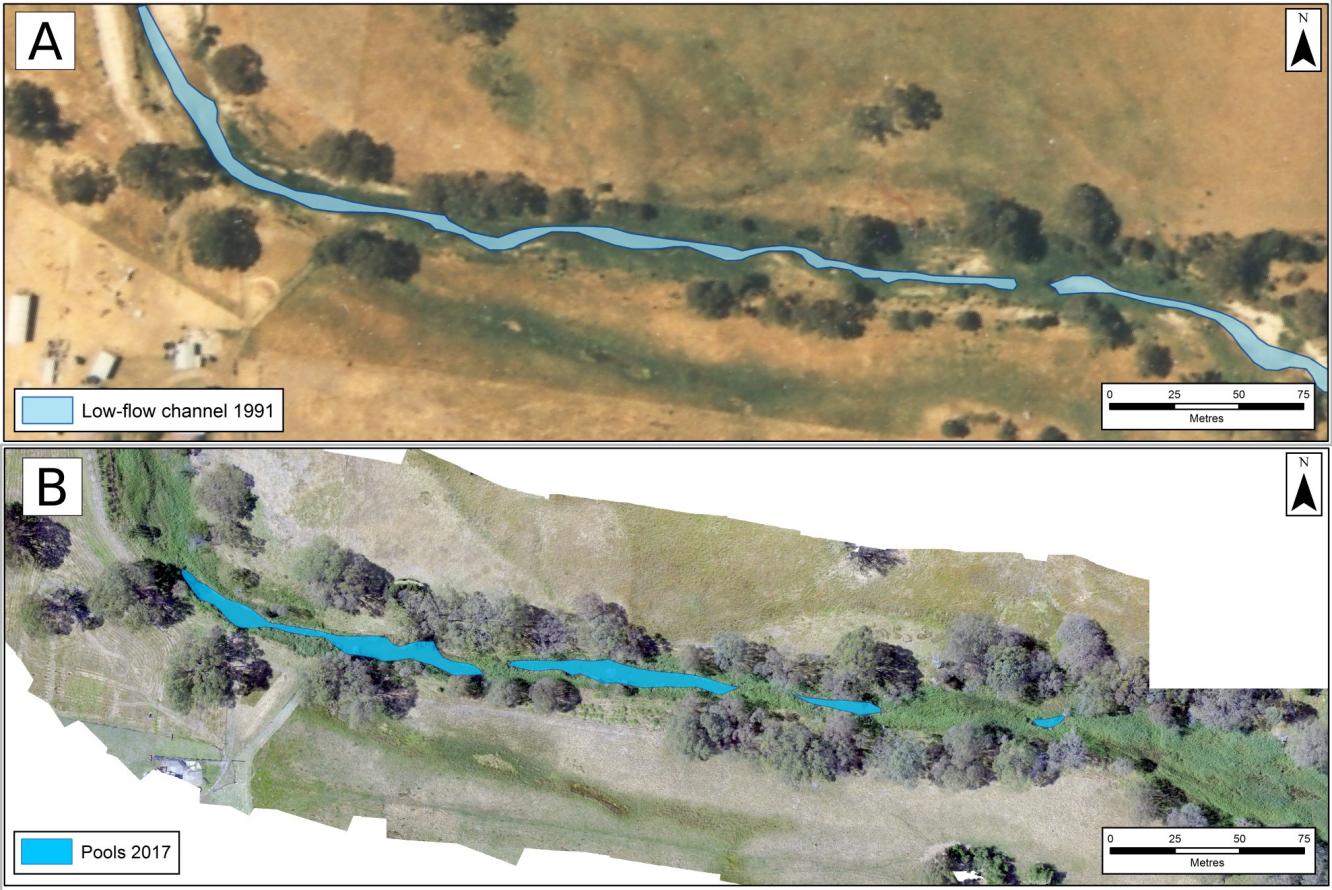
The transformation of reach 8, above Coleraine, from (A) a continuous low-flow channel in 1991 to (B) a sequence of pools by 2017. *Phragmites* spread inwards from the channel margins, pinching-off the low-flow channel.

Although stock was excluded from sand bed reaches at different times, all followed a similar pattern of change: a low-flow channel formed, emergent macrophytes colonised the channel margins and then spread across the channel, pinching off the low-flow channel (or surrounding historic extraction pits) and deep pools formed. This process accelerated in reaches 7, 9 and 10 in the late 1980s/early 1990s, once in-stream extraction from reach 7 had ceased (Fig 15).

The dissected morphology has emerged in all other sand bed reaches, wherever stock can access the bed. The association between continued stock access and the dissected morphology holds where no intervention was ever applied (reach 8) and in reaches previously subject to in-stream extraction (reaches 8 and 11).

Overall, the impact of stock exclusion and revegetation in sand bed reaches has been markedly different than in clay bed reaches. In sand bed reaches a sequence of pools has formed, but in clay bed reaches, a simple morphology has formed. In clay bed reaches morphology was similar between reaches subject to different interventions, and stock exclusion fencing alone was not associated with clear changes in form. In sand bed reaches the up and downstream boundaries of interventions demarcate sharp changes in form. Moving from upstream to downstream (reach 6 to 11), the post-intervention morphology abruptly changes from dissected (reach 6), to sequence of pools (reach 7), back to dissected (reach 8), back to sequence of pools (reach 9 and 10) and then back to dissected (reach 11).

### Transitions in morphology

**Fig 16:**
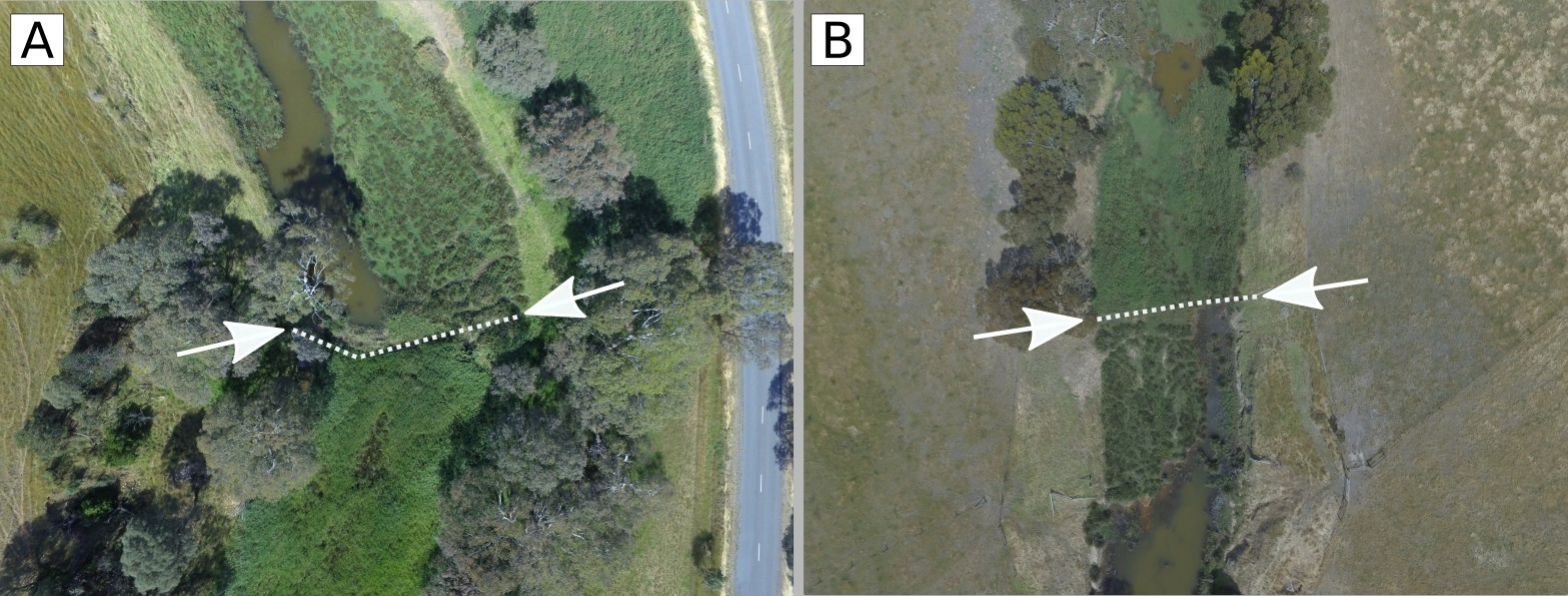
(A) The upstream boundary of reach 7 and 8 and the abrupt changes from *Juncus* (upstream, top of photo) to *Phragmites* (downstream, bottom of photo), (B) the downstream boundary of reaches 8 and 9 and the abrupt change from *Phragmites* (upstream, top of photo) back to *Juncus* (downstream, bottom of photo). White lines and arrows indicate the location of stock exclusion fences.

The boundary between different morphologies in sand bed reaches is sharp and is marked by an abrupt change from a dissected to a sequence of pools morphology (or vice versa) (Fig 16). The sharp boundaries occur at the upstream and downstream ends of stock exclusion and revegetation reaches, where a fence spans the river. Road crossings interrupt this pattern by suppressing all vegetation where they span the river, but *Phragmites* or *Juncus* (whichever was growing in the reach disturbed by the crossing) has established immediately downstream.

### Summary of results

The common feature that emerges from this complex series of changes is that sequence of pools morphology emerges whenever stock are excluded from a sand bed reach by a fence, even when the reach was earlier subject to in-stream extraction. The sequence of pools morphology is also associated with a large increase in the *Phragmites* cover between 1991 and 2017. The dissected morphology, associated with an increase in the coverage of *Juncus*, dominates sand bed reaches wherever stock access continues. The simple morphology, which includes some sand bed and clay bed reaches, is associated with continued stock access. The vector dispersion and std deviation of depths metrics show no consistent difference between reaches subjected to different interventions, but sum of squared height difference values are larger in reaches treated with stock exclusion and revegetation (where deep pools have formed).

## Discussion

Within the range of impacts that humans can have on stream systems, Bryan Creek is at the simple end of that range–an upstream source of sediment is delivered to a relatively uniform reach of river within a wide valley. Unlike many other streams in south east Australia, Bryan Creek has not been dammed, straightened or confined with artificial levees. However, like a multitude of other small streams across the world, in response to human disturbance of the catchment, it has enlarged, and aggraded with sand. Once the supply of sand from the catchment has declined, we would predict a reasonably orderly sequence of channel changes as the sand pulse moves downstream, with management interventions such as sand extraction, stock exclusion, and revegetation, introducing local perturbations to that sequence. In other words, we would expect the catchment scale sediment dynamics to determine the general patterns of geomorphic change in such a simple system. That orderly sequence is not what we have found on Bryan Creek. Instead, the patterns of change have been more complex than predicted, and apparently far more sensitive to local interventions. In particular, the system is extremely sensitive to in-stream vegetation, which in turn, is driven by whether stock can access the stream. This sensitivity is demonstrated by the sharp boundaries between reaches delineated by fences.

We discuss the scale of intervention and the crucial role that in-stream vegetation plays in shaping the morphology of sand bed reaches in more detail below.

### Predicted channel adjustment without intervention

In the absence of intervention, channel adjustments should track the downstream movement of the tail of the bedload pulse. That is, at any point in time, upstream reaches will have progressed further along their post-disturbance trajectory than those further downstream [38], and this is a classical example of an ergodic approach to stream management [39].

Development of Bryan Creek can be compared with this well described conceptual model of channel recovery following migration of a sand pulse. The model suggests that, as the tail passes downstream, bed variability increases and a sequence of channel adjustments takes place [40] including: channel contraction, bench formation (benches are stable bank-attached bars that accrete within the over-widened channel) and return of a well-defined low-flow channel [17, 41], exhumation of the pre-disturbance bed [42–44], stable alternating bars [45], establishment of in-stream vegetation [46, 47], and the re-emergence of pools [3, 40]. Alternatively, as the sediment supply declines, the river could also resume some other pre-pulse trajectory, such as incision (for example [20, 38]).

We expected that compared to the baseline thalweg profile (surveyed in 1984), the post-intervention thalweg profile (surveyed in 2017 and 2019) would be more variable upstream, in the tail of the sand wave, and variability would gradually decrease in a downstream direction.

### Predicted channel adjustment with intervention

Local scale interventions will accelerate this pattern of recovery by accelerating sediment removal (in-stream extraction), or by trapping and storing sediment (stock exclusion and revegetation). When superimposed on the tail of the bedload pulse, interventions would be expected to cause localised improvements in stream condition that slightly perturb the upstream-downstream pattern of channel adjustment i.e. the local interventions will play a secondary role to the catchment scale processes shaping the recovery of Bryan Creek.

Local scale interventions interrupt sediment continuity by either locking sediment into storage or removing sediment from storage, causing interventions in upstream reaches to interact with interventions further downstream. Recall too, that all of these interventions are superimposed on the tail end of a degrading sediment pulse. Further, changes are not restricted to simple erosion or deposition, but to more complicated geomorphic patterns and interactions with vegetation. The key point is that this conceptual model would predict that local scale interventions will interact with each other and with the catchment scale processes shaping Bryan Creek. These interactions will cause channel morphology to gradually change at the upstream and downstream end of intervention reaches.

### The impact of catchment vs. local scale intervention on reach morphology

Channel change in Bryan Creek has not followed the classical recovery trajectory described above. The overall morphology of Bryan Creek has been shaped by erosion and sediment production at the catchment scale [21]. However, rather than simply accelerating this pattern of morphological change (for example [48, 49]), local scale interventions have dramatically changed the underlying morphology of intervention reaches. In doing so, local interventions have been surprisingly effective (and varied) at shaping the physical structure of the river.

Our results demonstrate the challenge in predicting how a river will respond to local scale intervention when target reaches are impacted by processes operating at multiple scales, and when those processes are changing over time [50]. Catchment scale processes impact at the local scale by making a reach more or less sensitive to local scale interventions. In Bryan Creek, the sensitivity of a reach to intervention is determined by the passage of the sediment wave. Because each reach will be at a different stage of recovery at the time of intervention, and therefore be more or less sensitive to local scale interventions, the timing of interventions is as important as the spatial scale of those interventions [51]. Intervene too soon and the catchment scale processes will undermine the intervention (for example by burying vegetation with sediment), intervene too late and the catchment scale processes will make a reach insensitive to intervention (for example in the clay bed reaches of Bryan Creek).

### Channel recovery and geomorphic complexity

The complexity of the sequence of pool reaches in Bryan Creek is clearly greater than the flat sheets of sand they replace. The cross-section complexity of the incising, clay bed reaches are also more complex than the flat, clay bed that they replace. These differences in complexity emerge despite the fact that relative to the pre-intervention state, the clay bed reaches show little overall improvement in condition, while the sequence of pools reaches show a dramatic improvement in condition. The variability within treatment reaches of Bryan Creek is greater than the variability between treatment reaches of Bryan Creek. The fact that the range of variability values are indistinguishable between the different treatment types, even though the sequence of pools reaches are clearly of greater complexity and are in better geomorphic condition than both the dissected and simple morphologies indicates that cross-section and thalweg variability metrics serve as poor proxies for recovery in Bryan Creek. The type and character of variability that emerges at the tail of a bedload pulse following local scale intervention is better captured by morphological classification than by simple metrics. This demonstrates that as a river moves along a recovery trajectory, complexity may become a less meaningful surrogate for a more desirable, recovered morphology.

### Vegetation and morphology

In-stream vegetation plays a crucial role in the sand bed reaches of Bryan Creek, which alternate between dissected morphology and a sequence of pools morphology. The dissected morphology is associated with the invasive emergent macrophytes *Juncus acutus* [52], and the sequence of pools morphology with native *Phragmites australis*, which grows in wetlands, marshes and low energy rivers across eastern Australia [53]. These macrophytes influence morphology via their differing abilities to trap and store sediment.

When stock can access the river bed they feed on stands of *Phragmites* and trample emerging shoots. This enables dense mats of *Juncus*, which are otherwise out-competed by the tall, fast growing *Phragmites*, to establish on the river bed. *Juncus* reproduce by germination of flow dispersed seeds [54], which causes infestations to spread in a downstream direction. Once established, *Juncus* grows into strong tussock-like domes up to 1.5 m in height. The dense, spherical root mass of *Juncus* has a high tensile strength and resists uprooting during high flows, but the rooting depth is shallow (~0.2 m in depth). The physical characteristics of *Juncus* (salt tolerance, rapid dispersal, dense canopy, and a strong but shallow root mass) explain the dissected morphology in reaches 7, 9, and 12; *Juncus* act as erosion resistant projection above the ground, but are undermined by deep scour, which is concentrated into a network of low-flow channels between the clumps.

Where stock have been excluded from sand bed reaches (reaches 8, 10 and 11) *Phragmites* have colonised the bed and a sequence of pools has developed. Although pool-riffle sequences have re-emerged at the tail of a bedload pulse in gravel bed rivers [15, 55], or formed downstream of sediment retention structures in sediment laden rivers [56], both cases require that coarse sediment is exposed to initiate riffles. This is not the case in Bryan Creek where the bed consists of well sorted sand with underlying clay. The physical properties of *Phragmites* allow the sequence of pools to develop. Pre- and post-intervention surveys that were undertaken 23 years apart show that over this time pools have scoured, and the intervening bed, which is covered by *Phragmites*, has aggraded.

*Phragmites* can tolerate prolonged waterlogging, drought and moderate salinity levels [54]. *Phragmites* spread to downstream areas by dispersing seeds or rhizomes into the flow, and stands spread laterally by sending rhizomes into the surrounding sand [57]. These properties mean that *Phragmites* will spread through sand deposits quickly. Once established, *Phragmites* grow into tall (up to 4 m), dense stands that can impede flow and induce sediment deposition, but are flexible enough to survive higher flows [57]. The deep network of roots and rhizomes (up to 1 m deep) [58] increase cohesion and stabilise deposits, locking sediment into storage.

We propose that *Phragmites* have caused the sequence of pools to develop by trapping mobile sand, building benches on the channel margins, then spreading towards the channel centreline, aggrading the bed at the same time. Inspection of aerial photography from 1985, 1991 and 2010 revealed that as the *Phragmites* spreads across the channel, they pinch off the low-flow channel into a series of long, narrow pools. As they trap incoming sand, *Phragmites* expand further into the now isolated low-flow channel, but this encroachment is not uniform along the entire channel, causing the pools to shorten but also widen, whilst maintaining their position. In this manner, *Phragmites* behave as river system engineers [58], first building benches on the channel margin and then elevated ‘ledges’ that span the river between the pools.

The efficiency of *Phragmites* in trapping and storing sediment is supported by [59] who documented aggradation of gullies colonised by *Typha* and *Phragmites* in eastern Australia. Erskine *et al*. [16] also invoked the sand trapping and storing abilities of *Phragmites* to help explain channel contraction in Widden Brook, a sand bed stream in eastern Australia.

The upstream part of reach 11, which is dominated by *Juncus* but is downstream from reaches dominated by *Phragmites*, incised between 1984 and 2019. We believe that this incision was promoted by the trapping of sediment in upstream reaches. Incision at the downstream end of reach 11 is driven by an ongoing extraction within the Wannon River, which has locally increased channel grade, an adjustment prevented from migrating upstream into Bryan Creek by the road crossing at a chainage of 2 km.

### Intervention boundaries and interaction between reaches

Overall, Bryan Creek has not followed a typical recovery trajectory of a sand bed river impacted by a bedload pulse. Given that the boundary conditions of the different treatment reaches are the same, we would expect differences between reaches to gradually disappear with time. Instead, differences in morphology between reaches have been amplified with time. A surprising finding of this research is the abrupt transitions between dissected and sequence of pools morphology in sand bed reaches, and the apparent lack of interaction between reaches.

Unregulated rivers exhibit upstream-downstream continuity of sediment transport. We would expect the changes in sediment storage caused by local scale interventions, expressed as an increase or decrease in thalweg elevation, to generate changes in thalweg elevation in upstream and downstream reaches. Because the effect of a change in storage diminishes with distance from the perturbation [60], we expected smooth transitions in morphology: the opposite of the pattern observed in Bryan Creek.

**Fig 17:**
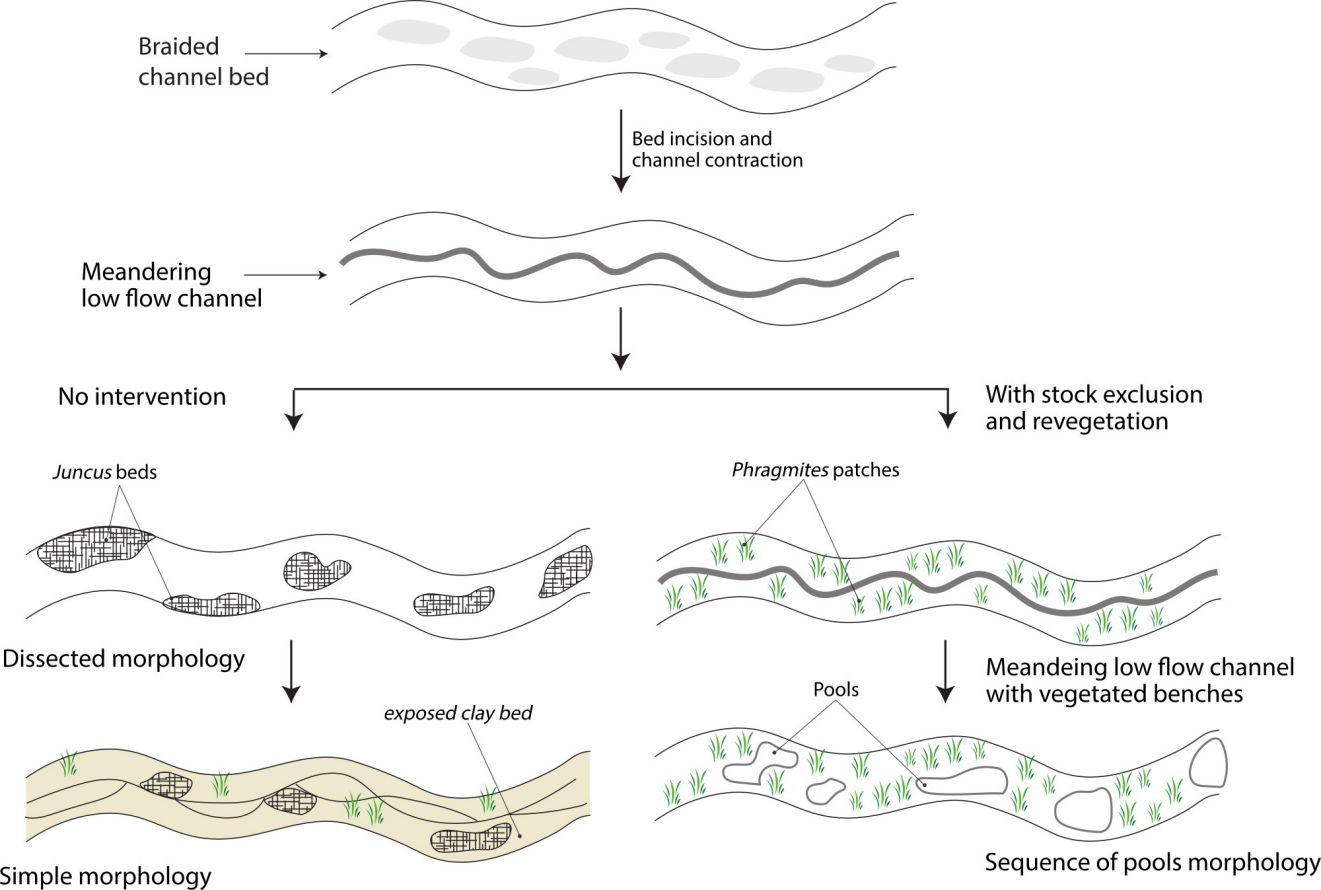
A revised conceptual model of the recovery of sand bed streams at the tail of a bedload pulse which have (or have not) been treated with stock exclusion fencing and bank revegetation at the local scale, following the earlier control on sediment supply from the catchment.

We propose that in reaches where stock have been excluded *Phragmites* has immobilised sediment, which has interrupted sediment continuity and disconnected reaches from each other (acting as barriers *sensu* Fryirs, [61, 62]). By drastically reducing the movement of sediment through stock exclusion reaches *Phragmites* supresses interaction between reaches. The important point here is that interaction between reaches is regarded as the major, and historically under-appreciated, factor controlling the outcome of local scale interventions [23, 63]. Our findings in Bryan Creek show that when catchment scale drivers of degradation have been addressed, local scale interventions that promote in-stream vegetation can disconnect reaches, prevent interaction and drive localised changes in geomorphic condition (Fig 17).

### Implications

The most important finding of this research is that local scale interventions can deliver substantial improvements in stream condition, at least when measured in terms of channel morphological complexity. This is especially true when sediment supply is declining at the tail of a sediment pulse. In Bryan Creek, rather than interacting to accelerate the orderly sequence of recovery from upstream to downstream, interventions interact very little, instead causing discrete changes in morphology that are separated by sharp boundaries. This suggests that once the catchment scale issues have been addressed, individual reaches can be highly sensitive to local scale interventions that can be exploited to substantially improve stream condition.

In-stream vegetation determines the morphology of different reaches, and the sharp transitions between them. The tendency for some species of emergent macrophytes (e.g. *Phragmites*) to trap sediment and promote pool development is crucial. These findings suggest that when intervening to improve stream condition managers should focus on excluding stock so that in-stream vegetation can establish. Furthermore, these efforts should be concentrated in reaches that still have mobile sand, as once the underlying bed is exposed, interventions have little impact.

This research also implies that, although in-stream sand extraction accelerates bed degradation at the tail of a sediment pulse, this degradation does not, on its own, accelerate geomorphic recovery. Instead, the trajectory of reaches subject to in-stream extraction depends on the local sediment supply, which in turn is altered by in-stream vegetation. This observation is useful because sand extraction is often proposed as a solution for sand pulses in streams [10].

## What is the generality of these results?

There is substantial literature that looks at the dynamics of sediment pulses moving through streams. Most of this literature concentrates on the dynamics of the wave front rather than on what happens once the wave has passed. Bartley and Rutherfurd [20] tested the proposition that the stream bed would return to its pre-pulse geomorphic diversity once the pulse had passed. This was borne out in the Ringarooma River in Tasmania. Two clear contrasts on Bryan Creek are, first, that without intervention the channel eroded to the clay. Second, with stock exclusion and with *Phragmites* invading the stream bed, the river developed complex morphology within a few decades. The contrast between the clay bed and the deep pools in this creek demonstrates the influence of local interventions on the recovery from a sand pulse. The fact that Bryan Creek is ephemeral, and that vegetation can invade the channel bed, make this class of stream particularly sensitive to local interventions.

## Conclusions

Most stream restoration activities take place on small agricultural streams. Such streams are often degraded by a typical suite of disturbances including riparian clearing, catchment disturbance and a consequent pulse of sediment. Once the supply of sediment from the catchment has been managed, the assumption is that sediment will pass through the stream as a classical wave, with the stream progressively recovering downstream as the wave translates. This conceptual model has provided a very useful way to think about where and when to intervene in attempts to restore such streams.

This paper has assessed the impact of local scale interventions at accelerating geomorphic recovery at the tail of such a bedload pulse in a small agricultural river, once sediment supply from the catchment has declined. This was done by classifying the changes in morphology observed in 11 distinct reaches of Bryan Creek, and by comparing the level of geomorphic complexity between reaches subject to three typical restoration interventions: sand extraction, stock exclusion fencing, and revegetation. The complex restoration activities on this stream, that vary over the space of hundreds of metres, are typical of the piecemeal character of many stream restoration projects.

The pattern of geomorphic recovery observed in Bryan Creek did not conform to our predictions of gradual improvements in stream condition from upstream to downstream. Cross-section variability did decrease in a downstream direction but was similar between all sand bed reaches. Thalweg variability did not gradually increase in a downstream direction but was spectacularly higher in reaches where stock had been excluded. Overall, local scale interventions appear to dominate over catchment scale processes in this stream.

Stock exclusion and revegetation did not lead to a meaningful improvement in stream condition in clay bed reaches, which have continued a trajectory of slow incision and simplification. In sand bed reaches morphology abruptly alternated between a degraded, dissected morphology and a sequence of pools. The boundary between the two morphologies was sharp, coinciding with stock exclusion fences that span the river, and suggesting little interaction between different interventions.

The trapping and storing of sediment by *Phragmites*, which grow in reaches where stock have been continuously excluded, and the undermining of *Juncus*, which grow in reaches that stock can access, explains the two very different morphologies.

We suggest that this research has the following implications for stream managers:

1. Local scale interventions can powerfully alter catchment scale processes.
2. Once the clay bed beneath a sediment pulse is exposed interventions have much less effect on stream condition and channel complexity. Thus, interventions should occur before the underlying clay bed is exposed, while there is still mobile sand left in the channel.
3. Stock exclusion is crucial as it allows emergent macrophytes (such as *Phragmites*) to establish on the bed, and for pools to develop. In this setting, *Phragmites* can cause pools to develop in as little as 5 to 7 years.
4. Riparian revegetation, in this situation, had little impact on channel complexity or overall stream condition when used without stock exclusion.

## Supporting information

S1 Table All 2019 cross sections. (XLSX)

S2 Table 1980 pre-intervention thalweg profile. (XLSX)

S3 Table Post-intervention variability metrics by chainage. (XLSX)
